# Human and Economic Cost of Disease Burden Due to Congenital Hypothyroidism in India: Too Little, but Not Too Late

**DOI:** 10.3389/fped.2022.788589

**Published:** 2022-05-03

**Authors:** Ramesh Vidavalur

**Affiliations:** Department of Neonatology, Cayuga Medical Center, Ithaca, NY, United States

**Keywords:** congenital hypothyroidism, newborn screening, economic evaluation, value of statistical life, disability adjusted life year, newborn, India, Benefit-Cost Analysis

## Abstract

**Background:**

Congenital hypothyroidism (CH) is one of the most common preventable causes of mental retardation. Implementing newborn screening (NBS) in >52 countries enabled early detection and to initiate treatment of neonates with CH. India is yet to implement a national NBS program even though an estimated 5–15% of sick newborns suffer from genetic and metabolic disorders. Recent pilot studies confirm that the CH incidence rates range from 1 in 500 to 1 in 3,400 live births. Our objective was to estimate overall incidence rates of congenital hypothyroidism and to evaluate the costs and benefits of implementing universal NBS for CH in India.

**Methods:**

We used the best available epidemiological and cost data to synthesize incidence rates and screening costs for CH in India. We conducted a meta-analysis of country-specific published literature and included 14 studies to calculate baseline CH incidence rates. We used two models to estimate intellectual disability in unscreened cohorts. Disability-adjusted life years (DALY) were calculated to quantify burden of disease utilizing disability weights. Direct costs including screening, confirmatory tests, and treatment costs were obtained from public and private market sources. Economic benefits were calculated from lost DALY using human capital approach and value of statistical life methods, utilizing gross national income (GNI) per capita data and value of statistical life year (VSLY), respectively. Cost discounting was used to estimate the present value of future benefits over lifetime of affected newborns.

**Results:**

The incidence rate of CH in India is 72 (95% CI: 58, 85) cases per 100,000 live births. Based on this data, 1 in 1,388 (95% CI: 1166, 1714) infants were diagnosed with CH in India for the year 2018. The estimated annual incidence ranged from 14,000 to 20,730 cases, and those at risk for intellectual disability ranged from 5,397 to 13,929 cases. Estimated discounted and undiscounted lost DALYs were 57,640 and 410,000, respectively. Direct annual costs for universal screening for CH in India is around USD187 million. Based on current incidence and expected severity of sequelae, economic losses ranged from USD 159 million to 1.1 billion. Benefit–cost ratios ranged from 1.8 to 6.

**Conclusions:**

Universal NBS for CH is one of the healthcare interventions that is beneficial to prevent morbidity and cost saving. The cumulative economic benefits, derived from prevention of intellectual disability, assuming cost effectiveness threshold of three times of gross domestic product per capita, far outweigh the direct and indirect costs of screening, treatment, and surveillance throughout the life of the affected individuals. Our analysis strongly supports the argument for investing in NBS that provides good value for money and would yield substantial financial gains for the country.

## Introduction

Congenital hypothyroidism (CH) is the most common preventable cause of intellectual disability worldwide (1, 2). Primary congenital hypothyroidism is the result of developmental defects of the thyroid glands, mainly due to thyroid agenesis or dysgenesis or dyshormonogenesis that can lead to severe acute and chronic clinical symptoms including long-term intellectual impairment. Neonatal screening for CH, first implemented almost half a century ago, offers a window of opportunity for timely diagnosis of CH, to initiate appropriate treatment and prevent long-term morbidity. It is estimated that 7 in 10 newborns with CH are born in areas that have no neonatal screening programs (3).

Most of the industrialized nations have implemented healthcare system changes to incorporate newborn screening in the last five decades to detect early, treat promptly, and eliminate neurodevelopmental impairment from CH (4). Unfortunately, majority of nations with the highest burden of CH does not have effective, established universal newborn screening programs to eliminate disease burden. This poses considerable public health challenge to limit preventable chronic morbidity in population (3), as it is one of the most common causes of cognitive impairment in newborns that has an enormous societal impact if screening is not done and replacement therapies are not initiated in timely manner (5). Early detection and prompt treatment of CH (within the first 2 weeks of life) are essential to optimize the neurocognitive outcome, linear growth, the onset and progression of puberty, pubertal growth, and final height of affected neonates (6).

**Figure 1:**
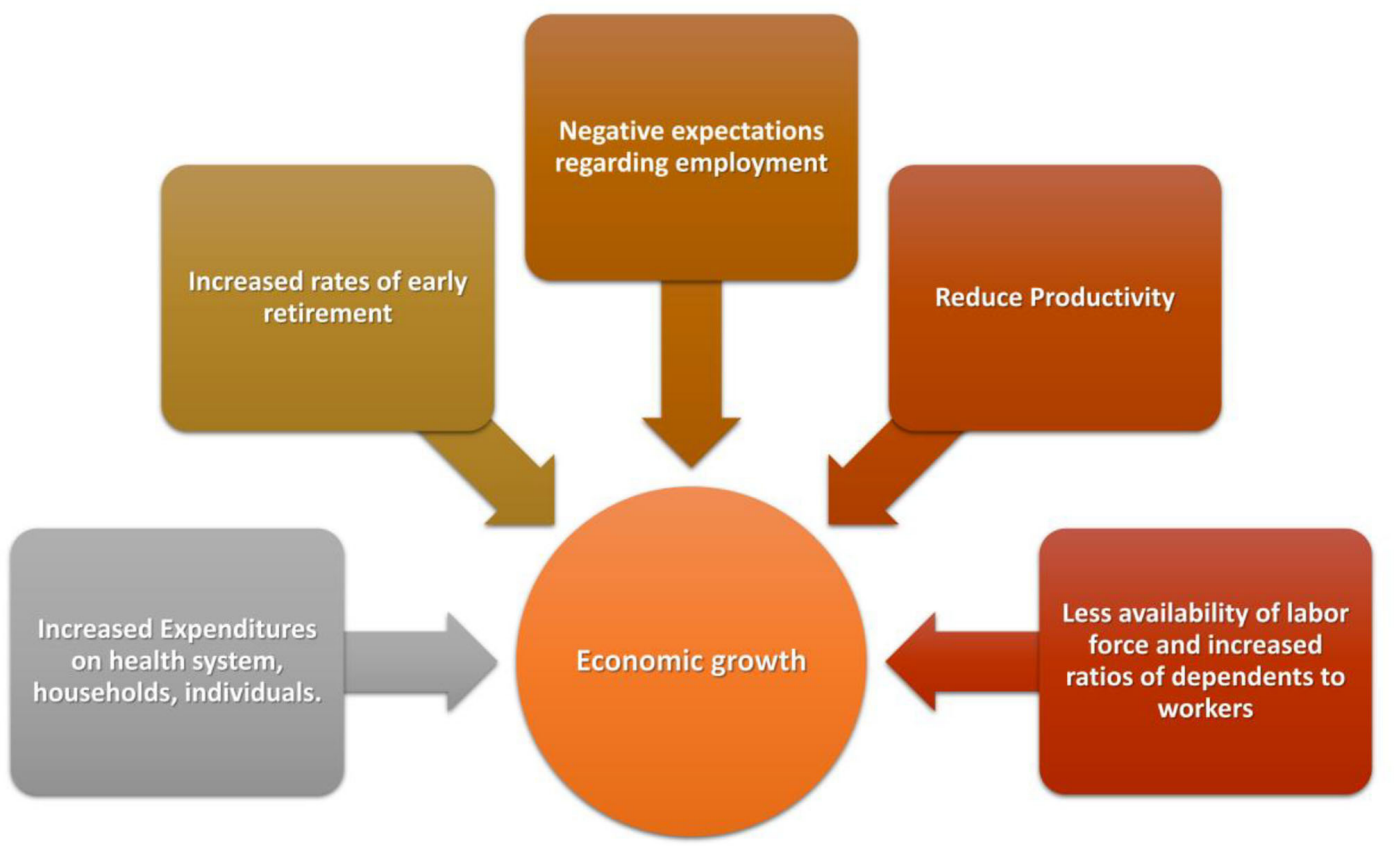
Effects of poor health on economy.

Moreover, morbidity from noncommunicable diseases is increasingly recognized to have sizeable economic impact on households, industries, and societies, both via the consumption of health services and via losses in income, productivity and human capital (Figure 1). Fortunately, in the past two decades, early initiation of treatment and improvement in the overall management of CH patients have resulted in better cognitive and motor developmental outcomes, comparable with those of controls (7).

Historically, all the newborn screening programs in high-income countries are implemented as a part of public health mandated programs with centralized facilities for testing, reporting, and surveillance. From welfare economics' point of view, one of the main reasons for countries to do this is the belief that early detection of a few disorders can offer societal health benefits by mitigating or reducing healthcare burden on worse outcomes of these disorders. Selecting these disorders for national newborn screening panels is based on several factors—cost of screening, feasibility, and extent of health benefits.

With >25 million annual births and prevalence of CH varying between 1 in 600 to 1 in 1,700 newborns (8–11), India screens <2% of newborns for CH annually (12, 13). Currently, only 5 out of 26 states have started, or are in the process of, establishing universal screening programs to screen for CH. In addition, the World Health Organization recommends introduction of genetic services in countries with infant mortality rates of <40 per 1,000 live births. As India is striving to achieve Sustainable Development Goals (SDG), especially to keep neonatal mortality rate under 12 per 1,000 live births with the introduction of the India Newborn Action Plan within the framework of Janani Suraksha Yojna and other national health mission programs, time is ripe to introduce programs to reduce disease burden of noncommunicable diseases and other nonfatal risks in newborns (14).

To make public healthcare decisions at the policy level, it is essential to have a detailed analysis on cost savings, cost effectiveness, or cost benefit of necessary public programs. Many of these analyses are based on multiple assumptions. It not only requires frequency of health conditions along with various outcomes in the context of with or without interventions but also need to take economic and opportunity costs into consideration. Thus, quantifying the health and economic benefits of population-level screening program, especially in newborns, is challenging. While India's MDG and SDG commitments to reduce infant mortality have been substantially successful, there is an urgent need for renewed focus on morbidity-preventing strategies, such as universal newborn screening for common conditions that poses considerable long-term health risks. Countrywide screening programs, if implemented efficiently in a populous country like India, can provide safety nets, minimize adverse outcomes in relatively modest number of disease-afflicted cases, encompassing overall improvement in societal health. At the same time, these programs should also be thoroughly evaluated beforehand to estimate projected net health benefits in order to invest in strong health systems that prioritize newborns with appropriate healthcare resource allocation.

The purpose of this study was to estimate the burden of disease, assess economic losses from reduced human capital secondary to disease-associated morbidity, and to examine benefits and costs from universal newborn screening program for CH in India. These data will inform country-specific estimates of CH in India and allow prioritization of resource allocations toward child health.

## Methods

### Design and Population

Estimates of congenital hypothyroidism in India: Literature searches of electronic databases including PubMed, OVID, and Google Scholar were performed using a combination of search terms: (((((Congenital hypothyroidism) AND (India))) AND (prevalence)) OR (incidence)) AND (newborn screening)). Of 13,330 studies identified by our initial search, we selected 178 publications that were relevant to our study. Among them, we identified 14 studies that provided incidence data. We excluded studies that had <500 subjects to minimize heterogeneity and to maximize precision and accuracy. We performed a random effects meta-analysis using standard techniques to calculate pooled estimates with 95% confidence intervals. We assessed heterogeneity using I^2^ and publication bias with Eggers test. Statistical significance was set at *p* < 0.05. For population parameters, including population, births, life expectancy, and GNI, relevant sources are shown in Table 1.

**Table 1:** Input parameter pooled estimate for incidence of hypothyroidism in India.

**Input parameters**		**Lower**	**Upper**	**References**
**Number of Births**	24,164,360			MOHFW, GOI
**Incidence of CH**	0.000720	0.000583	0.000857	Point estimate from our study
**Frequency of Sequelae-Model 1**				Barden et al. (15)
Mild ID	0.4			
Moderate ID	0.25			
Severe ID	0.15			
**Frequency of Sequelae-Model 2**				ACMG, Committee on Genetics (16)
Mild ID	0.27			
Moderate ID	0.021			
Severe ID	0.019			
**Disability weights**				Mathers et al. (17)
Mild ID	0.29			
Moderate ID	0.43			
Severe ID	0.82			
**GNI per capita**	$6,427			World Bank (PPP method)
**Costs**				
Screening Costs	$173,500,105			Our estimates
Confirmation Assay cost	$125,020	$101,196	$148,843	
Medication Costs:	$8,501,347	$6,881,341	$10,121,354	
Follow up Costs				
Physician visits	$4,000,634	$3,238,278	$4,762,990	
Laboratory costs	$1,500,238	$1,214,354	$1,786,121	

*ID-Intellectual Disability; Costs converted from INR to 2018 USD*.

### Years of Life Lost, Years Lost Due to Disability, Disability-Adjusted Life Years

The disability-adjusted life years (DALY) is defined as the sum of years of life lost (YLL) and years lost due to disability (YLD) (i.e., DALY = YLL + YLD) (18, 19). YLL was not calculated in this study as mortality is uncommon in cases with CH.

To perform a patient-focused analysis including the lifetime impact of congenital hypothyroidism on DALY, the morbidities in terms of intellectual disability anticipated with degree of severity of hypothyroidism were included in the calculation of YLD. Specifically, YLD was calculated by multiplying the incidence (I) of intellectual disability due to untreated congenital hypothyroidism by a disability weight (DW) (17) associated with the degree of disability and the anticipated duration of that state with intellectual disability (L) (i.e., YLD = I × DW × L). The DW was included in the calculation of the YLD account for severity of disease and disability. They range from 0 (perfect health) to 1 (worst possible health state). Frequencies of mild, moderate, and severe intellectual disability (ID) from CH and associated disability weights were obtained from existing literature (15, 16), as the accurate prevalence and projection of long-term outcomes of overt disabilities (intellectual or physical or behavioral) at population level, in the absence of established CH screening program, is difficult to capture and prone to have substantial variations as most of existing prevalence and outcome estimates come from published clinical case series literature. To quantify the magnitude of outcomes in the absence of screening, we developed two models—Model 1 and Model 2. Model 1 is based on the frequency of intellectual disability due to CH from previous economic evaluations from the United States and Australia (15, 47) where they followed evidence from clinical case series in the prescreening era. Model 2 is based on the American College of Medical Genetics (ACMG) committee estimates of intellectual impairments, used in cost utility analysis of newborn screening programs in the United States (19, 48). We used country-specific life expectancy assuming constant disability from undiagnosed and untreated CH for the lifetime of the neonate. In summary, one DALY is equivalent to loss of 1 year of healthy life.

The notation “DALYs (r,K,ß)” is used to describe the results: where r = discount rate, K = age-weighting modulation factor, and ß = parameter from the age-weighting function. DALYs estimates were presented with [DALYs (0.03, 1., 0.04)] and without [DALYs (0,0,0.04)] age weighting and discounting (18).

### Costs

We included screening, confirmation, treatment costs from industry sources, academicians, and the Government of India website (http://www.nppaindia.nic.in/). Cost data are presented in the Supplementary Information. Costs for screening tests were obtained from two resources—Medplus Labs and Genes n' Life labs, two private sector labs. These costs were cross checked with nationally funded pilot project costs and also correlated well with inflation-adjusted costs for screening in the United States and recent region-specific literature (20, 21). Screening costs included supplies for sample collection, consumables, logistics, assay, labor, overhead costs, and confirmation assay. Clinical care costs included annual physician visits, annual laboratory testing, and medications. Frequency of testing and physician visits were modeled as per clinical practice guidelines recommended by the Indian Society of Pediatric and Adolescent Endocrinology for newborn screening for primary CH (22, 23).

### Economic Value of Lost Disability-Adjusted Life Years

#### Human Capital Approach

Using the human capita (HC) approach, we estimated lost productivity from three values—number of disabilities, number of DALY losses, and gross national income per capita (GNI) (24). GNI per capita is an estimate of average individual productivity to the national economic output in a given year. Monetizing the number of DALYs lost from a specific cause can be used as proxy for non-productive years from an individual perspective that reflects the lost opportunity of significant domestic productivity of a developing country. DALY losses with 95% uncertainty levels were used in the calculations, and sensitivity analysis was done with different discount rates (3%, 5%, 7%) and assumed reductions in morbidity to estimate the present value of future benefits. We modeled these estimates as follows:

Based on the total number of disabilities, YLD lost was calculated using a life expectancy of 67 years. We used GNI per capita (PPP—*purchasing power parity* method) to estimate the total economic value of estimated disabilities. Economic losses were calculated from monetizing lost DALYs and multiplying these life years with GNI per capita. Based on clinical evidence, we assumed that there would be no mortality from CH (YLL), and total YLD reflect total DALY (Table 3).


$$\begin{array}{l} \text{Economic productivity loss}=\text{DALY lost}\times \text{GNI per capital}\\ \;\times \text{discount factor} \end{array}$$

#### Value of Statistical Life Approach

The value of monetized DALY is based on the value of statistical life year (VSLY), derived from VSL as described elsewhere (25). VSL reflects individual willingness to pay (WTP) for a reduction in mortality and morbidity, and we followed recent reference case benefit cost analysis guidelines to calculate the value of morbidity reductions (26). We used this method to determine ranges of three VSL estimates in a standardized sensitivity analysis as follows:

Step 1. First estimate calculated by multiplying GNI per capita by a factor 160;

Step 2. Multiplying GNI per capita by a factor of 100;

Step 3. Extrapolate VSL from a US estimate to India using an elasticity of 1.5 using the formula: VSL^India^ = VSL^USA^ × (GNI^India^/GNI ^USA^)^1.5^

Then, we obtained VSLY, which mirrors and individual's WTP. A constant VSLY was derived from a standard, population-averaged, country-specific VSL estimate by dividing life expectancy at the average adult population age, which is equivalent to one half of life expectancy at birth as a rough proxy. This constant VSLY is considered as an indirect estimate of monetized lifetime value of a DALY. Finally, we measured the lifetime economic productivity gains by multiplying VSLY with the number of lost DALYs and sensitivity analysis done for estimated reductions in morbidity (Table 4). All costs and benefits are presented in United States Dollars (USD).

## Results

### Prevalence of Congenital Hypothyroidism and Its Sequelae

Our random effects meta-analyses (Table 2) show the pooled estimate of incidence of congenital hypothyroidism in newborns at 1 in 1,387 (95% CI: 1 in 1,165, 1 in 1,714). This estimate translated to 72 (95% CI: 58, 85) cases per 100,000 live births. There was marked heterogeneity in incidence of CH in different geographical regions among the included studies (I^2^ = 56%). We determined that, out of 26 million births in the year 2018, 17,412 (95% CI: 14,094, 20,730) infants were expected to be diagnosed with CH. Among those diagnosed with CH, if untreated, the number of infants at risk for intellectual disability from Model 1 and Model 2 were 13,929 newborns (range: 11,275–16,584) and 5,397 (range 4,369–6426), respectively (Table 3).

**Table 2:** Pooled estimate for incidence of hypothyroidism in India.

**References (8, 10, 27–33)**	**Incidence**		
	**1 in**		
		**95% CI**	
Desai et al. (27)	2,481	1,322	20,095
Desai et al. (28)	2,804	1,696	8,093
Rama Devi and Naushad, (29)	1,700	1,064	4,223
Sanghvi and Dewakar, (10)	500	275	2,749
Kishore et al., (30)	1,042	719	1,893
Sudha et al., (31)	900	673	1,357
Kaur et al. (32)	1,400	959	2,594
ICMR Chennai (34)	727	531	1,155
ICMR Delhi (34)	1,141	793	2,032
ICMR Hyderabad (34)	1,383	918	2,800
ICMR Kolkata (34)	1,255	842	2,460
ICMR Mumbai (34)	1,544	1,000	3,382
Verma et al. (8)	1,706	1,429	2,117
Verma et al. (33)	1,486	899	4,288
			
Point Estimate with 95% CI (I^2^ = 56%)	1,388	1,166	1,714

**Table 3:** Severity of congenital hypothyroidism (CH) morbidity.

**Severity of CH**	**Expected No of Infants with Sequelae (Range)**	
	**Model 1**	**Model 2**
Mild ID	6,964 (5,637–8,292)	4,701 (3,805–5,597)
Moderate ID	4,354 (3,523–5,182)	365 (295–435)
Severe ID	2,611 (2,114–3,109)	330 (267–393)
**Total**	**13,929 (11,275**–**16,584)**	**5,397 (4,369**–**6,426)**

*ID, Intellectual Disability*.

### Estimated Disability-Adjusted Life Years Loss

Considering the average estimate of affected CH cases with neurological sequelae, the net DALYs lost in Model 1, with and without discounting or age weighting were 194,076 and 410,267 respectively. Model 2 estimates ranged from 57, 640 and 121, 849 with and without discounting, respectively (Table 4).

**Table 4:** Lost disability adjusted life years (DALYs) from CH related intellectual disability.

		**Mild ID**	**Moderate ID**	**Severe ID**	**Total**
Model 1	Lost DALY (0,0,0.4)^*^	137,348 (111, 175-163, 520)	127,283 (103, 028-151, 538)	145,636 (117,884-173,388)	410, 267 (332,087-488,446)
	Lost DALY (0.03,1,0.04)^*^	64,972 (52,591-77, 353)	60,211 (48,739-71, 685)	68,893 (55,765-82,021)	194,076 (157,093-231,059)
Model 2	Lost DALY (0,0,0.4)^*^	92,710 (75,043-110,376)	10,692 (8,654-12,729)	18,447 (14,932-21,962)	121,849 (98,629-145,067)
	Lost DALY (0.03,1,0.04)^*^	43,856 (35,499-52,213)	5,058 (4,094-6,022)	8,726 (7,064-10,389)	57,640 (46,657-68,624)

* *Disability-adjusted life year representation DALY (r,K,b) is used to describe the results where r, discount rate; K, age weighting modulation factor; b, parameter from age-weighting function; Ranges were given in brackets; ID, Intellectual disability; CH, Congenital hypothyroidism*.

### Cost Per Case Detected

At incidences of 1:1,165, 1:1,387, and 1:1,714, the cost to detect each primary CH case is $9,050, $10,775, and $13,312, respectively. For countrywide CH screening and subsequent management, it costs approximately $187 million (range $184–$190 million) annually.

### Economic Benefits

Using the HC approach, Model 1 and Model 2 estimates of the economic benefits of reducing undiscounted DALYs lost due to CH were $1.1 billion (range: $917 million to $1.3 billion) and $336 million ($272 million to $400 million), respectively. With discounting, they ranged from $434 to $638 million and $128 to 189 million (Table 5). Using standardized sensitivity analysis, values of VSL ranged from $347,634 to $1.02 million, and values of VSLY estimates ranged from USD $5,189 to $15,348 (Table 6).

**Table 5:** Estimated lost economic productivity—human capital method.

			**Lost Productivity in USD (Range)**		
		**DALYs (Range)**	**3%**	**5%**	**7%**
Model 1	DALYs (0,0,0.4)	410,267 (332,087–488,446)	$1,133,817,984 (917,758,954–1,349,874,250)	$788,300,083 (597,610,482–878,987,884)	$553,725,062 (448,207,861–659,240,913)
	Benefit-Cost Ratio		6 (4.8–7.1)	3.9 (3.1–4.6)	2.9 (2.3–3.5)
	DALYs (0.03,1,0.04)	194,076 (157,093–231,059)	$536,350,374 (434,143,786–638,556,963)	$349,251,407 (282,698,279–415,804,534)	$261,938,555 (212,023,709–311,853,401)
	Benefit-Cost Ratio		2.8 (2.3–3.4)	1.8 (1.5–2.2)	1.4 (1.1–1.6)
Model 2	DALYs (0,0,0.4)	121,849 (98,629–145,067)	$336,743,114 (272,572,090– 400,908,611)	$219,274,586 (177,488,803– 261,056,770)	$164,455,939 (133,116,602– 195,792,577)
	Benefit-Cost Ratio		1.8 (1.4–2.1)	1.1 (0.94–1.4)	0.87 (0.7–1)
	DALYs (0.03,1,0.04)	57,640 (46,657–68,624)	$159,294,480 (128,941,751– 189,649,972)	$103,726,638 (83,962,070– 123,493,005)	$77,794,978 (62,971,553– 92,619,754)
	Benefit-Cost Ratio		0.86 (0.68–1)	0.56 (0.44–0.65)	0.42 (0.33–0.49)

**Table 6:** Estimated lost economic productivity—value of statistical life method.

			**Model 1**			**Model 2**		
			**Estimated Loss of Productivity** ^*^		**Benefit-Cost**	**Estimated Loss of Productivity** ^*^		**Benefit-Cost**
			**in USD (Range)**		**Ratio**	**in USD (Range)**		**Ratio**
	**VSL** ^a^	**VSLY** ^b^			**Range**			**Range**
			**DALYs (0.0.0.4)**	**DALYs (0.3, 1, 0.04)**		**DALYs (0.0.0.4)**	**DALYs (0.3, 1, 0.04)**	
**VSL**_**160**_	1,028,320	30,696	$12,593,604,819 (10,193,782,204–14,993,396,738)	$5,957,380,069 (4,822,145,485–7,092,614,653)	31.7–67.1	$3,740,291,453 (3,027,527,561–4,452,993,953)	$1,769,324,322 (1,432,188,843–2,106,490,498)	9.4–19.9
**VSL**_**100**_	642,700	19,185	$7,871,003,012 (6,371,113,878–9,370,872,961)	$3,723,362,543 (3,013,840,928–4,432,884,158)	19.8–41.9	$2,337,682,158 (1,892,204,725–2,783,121,221)	$1,105,827,701 (895,118,027–1,316,556,561)	5.8–12.4
**VSL USA** ${}_{\text{1.5}}^{\text{c}}$	347,634	10,377	$4,257,397,850 (3,446,113,092–5,068,672,232)	$2,013,953,706 (1,630,175,960–2,397,731,453)	10.7–22.6	$1,264,444,059 (1,023,486,882–1,505,380,481)	$598,138,315 (484,166,193–712,120,814)	3.1–6.7

a *Value of statistical life (VSL): Country level population average value of VSL estimate using GNI per capita and assumed income elasticity-based on GNI- India: $6,427(2018); GNI-USA: $57,900 (2017)*.

b *Value of statistical life year (VSLY): A constant VSLY averages health status over lifetime*.

*^c^This VSL is extrapolated from US estimate to India using an income elasticity of 1.5. This option is preferred as it takes the efforts taken to reduce mortality and morbidity risk in low and middle income countries*.

* *Productivity calculated from monetized DALYs using VSLY over life time*.

Estimated net monetary benefits were $348 million (range $249–448 million) and $819 million (range 639–1 billion) from HC and VSL methods, respectively. Using the same econometric methods, benefit–cost ratios from HC methods ranged from 2.8 (95% CI: 2.3, 3.4) to 6 (95% CI: 4.8,7.1) when evaluated with discounted benefits and costs at 3% over a lifetime. Benefit–cost ratios were much higher ranging from 10.7 to 31.7 when VSL methodology was used to monetize lost DALYs. Overall, our analysis revealed newborn screening costs $457 (range: $389–$556) per DALY averted.

## Discussion

This study confirms pooled prevalence rate of CH at 7 per 10,000 live births, comparable with other subnational studies and worldwide prevalence rates. Our population model predicts the present value of future benefits, if uniform national screening, implemented at an expense of $187 million, outweighs the costs incurred. We estimate that the costs incurred to avert loss of a DALY remain at $457–$966, which is less than the one-time GDP per capita and cost effective as per WHO—CHOICE guidelines (35).

Now in the sixth decade, universal newborn screening for different disorders reduced significant disease burden in terms of morbidity and mortality worldwide. In the last decade, after extensive preparatory and execution phases to test the feasibility and collect data after newborn screening across five regions covering >100,000 newborns, a large collaborative study (34) from the Indian Council of Medical Research has recommended universal newborn screening for CH with an estimated overall prevalence of 1 in 1,130 newborns. The same study concluded that there were significant differences in prevalence of CH among different regions and early identification followed by subsequent timely thyroid replacement therapy resulting in better developmental quotients and growth velocity.

To further support timing and initiation of therapy and the critical role of thyroid hormone on normal brain development and function, few other clinical case series (36, 37) showed that burden of disease depends on time of diagnosis and treatment even after achieving euthyroid status once the diagnosis has been made. They have shown that infants who had delayed diagnosis and treatment had lower intelligent quotient (IQ) scores, scholastic performance, and behavioral problems. In contrast, a study from South Korea concluded that IQ scores, measured by the Weschler Intelligence Scale, were within normal limits when treatment started within 2–8 weeks of diagnosis of CH. Several regional retrospective studies from India have confirmed, in the absence of universal newborn screening, the average age of diagnosis is between 3 and 5 years, and the main reasons are lack of awareness among parents, community, and even among primary care physicians, which can lead to permanent sequelae of CH (38–40). Even if treatment for CH is started before 3 months of age, but later than 1 month, to prevent loss of IQ, many of these infants show some degree of impairment in school performance, speech, and fine motor skills later (41). A recent Irish longitudinal study (42) showed that rates of hypothyroidism in infants that were treated were 1 per 1,000 live births. If we apply that rate to 26 million births in India, we can expect around 26,000 infants that would need treatment, which is within the range of our modeling estimates.

Another recent Government-sponsored national household survey of persons with disabilities in India noted that the number of persons with onset of disability were 86 per 100,000 population, and applying this rate to a population of 1.35 billion, India suffers from significantly lower labor force participation rates, lower worker population ratio, and higher costs of disability support (43). As CH impairs optimal human development, economic losses from decreased labor productivity could be even larger than estimated. It is estimated that each one-point drop in IQ is estimated to reduce lifetime earnings by 1% (44). One study from Sweden found that even in cases of subclinical CH, testing showed an average IQ decrease by 7 points (45). Furthermore, a case series published from a tertiary referral center at the dawn of newborn screening confirmed that 65% of CH patients had an IQ of <85 and another 19% showed profound intellectual disability (46). Multiple large epidemiological follow-up studies concluded that earlier identification and treatment results in better neurodevelopmental outcomes.

Considering the history of newborn screening, a recent review (3) noted that only 29.3% of global births were screened for CH, and majority of the nonscreened infants are born in countries lacking nationwide newborn screening programs. The same study estimates that, across Asia, around 29,480 CH cases miss an opportunity of early screening and related benefits. If we consider these regional estimates, India accounts for >50% of affected newborns.

Poor health among children has remained too high for too long, despite decades of declaring agreements to reduce the burden by committing investments in maternal and newborn health. The newborn screening program promises to help in reducing health inequality, improve productivity, increase family savings, and strengthen the national economy. Saving one DALY through newborn screening costs less than $1,000, which is much smaller than VSLY and costs a fraction of gross domestic product per capita in India. Our analysis shows, even with minimal estimated benefits by discounting, that if we can reduce estimated DALY loss by 40%, implementing newborn screening for CH is cost effective.

### Strengths of the Study

To our knowledge, this is the first study to estimate and quantify the burden of congenital hypothyroidism in India. Given the resource restraints, we sought to understand the returns better in terms of health gains if we find resources to implement newborn screening and empirical benefit in preventing a proportion of disabilities caused by CH. For this, we followed three steps. First, we tried to quantify the burden of CH in terms of DALYs for births in year 2018. Over the last 2 decades, DALYs have been used as a key metric in estimating disease burden and monitoring public health. Second, we calculated the relative costs of implementing a comprehensive screening program, and finally, we analyzed the monetary value of avoided disability as the discounted present value of future earnings over their lifetime using various economic methods.

### Limitations of the Study

As with all cost-effectiveness studies for any healthcare interventions, our results are limited by available evidence regarding prevalence, frequency of morbidity, screening costs, and quality of life. Another challenge to estimate accurate benefits of NBS is lack of data regarding willingness to pay for health gains, country-specific utility or disability weights, accurate frequency of adverse health outcomes in the absence of screening, and absence of reliable population-based data on late diagnosis and treatment rates. We tried to minimize bias as much as possible by incorporating existing evidence, established estimates, and conducted sensitivity analysis. Costs for radio isotope imaging were not included as there is lack of universal availability and affordability, especially in rural areas. Indirect costs, such as lost parental income and disability care costs, were also not included due to paucity of data and lack of published evidence. General challenges include lack of public awareness and evidence of uptake rates even if universal newborn screening for CH is offered countrywide. Specific challenges include building core infrastructure, enhancing public laboratory and data systems, close surveillance, and follow-up systems to optimize outcomes.

## Conclusions

CH is one of the leading causes of intellectual disability in India. Newborn screening for CH in India seems to be one of the precious healthcare intervention programs that could be beneficial to every newborn, parent, community, and society-at-large. The impact of early identification and follow-up management in preventing lifelong morbidities for CH is undisputable. Our analysis strongly supports the argument of investing in NBS programs that provide good value for money and would yield substantial financial gains for the country. In addition to being cost effective and cost saving in the long term, broader implementation of screening programs might lay a strong foundation for better, healthy future for next generation, and maintain stronger productive workforce for sustainable development of better India. As a few states started implementing newborn screening for CH, it is time to focus on adopting universal newborn screening public health policy and needed resources. Coordinated efforts between healthcare professionals, policymakers, parents, and other stakeholders, including public–private partnerships, may help in building a lasting and successful newborn screening program in India.

## Data Availability Statement

The original contributions presented in the study are included in the article/Supplementary Material, further inquiries can be directed to the corresponding author.

## Author Contributions

RV conceptualized the study, collected the data, carried out the initial analysis, drafted the initial manuscript, reviewed and revised final manuscript, and agreed to be accountable for all aspects of the work.

## Conflict of Interest

The author declares that the research was conducted in the absence of any commercial or financial relationships that could be construed as a potential conflict of interest.

## Publisher's Note

All claims expressed in this article are solely those of the authors and do not necessarily represent those of their affiliated organizations, or those of the publisher, the editors and the reviewers. Any product that may be evaluated in this article, or claim that may be made by its manufacturer, is not guaranteed or endorsed by the publisher.

## Abbreviations

CI: confidence interval
CH: congenital hypothyroidism deficiency
DALY: disability-adjusted life year
GNI: gross national income per capita
VSL: value per statistical life
VSLY: value per statistical life year
YLL: years of life lost
YLD: years lived with disability.
