# Supplementary material for: Human and Economic Cost of Disease Burden Due to Congenital Hypothyroidism in India: Too Little, but Not Too Late
Source: Front Pediatr. 2022 May 3;10:788589. doi: 10.3389/fped.2022.788589 (PMC9110855; doi:10.3389/fped.2022.788589)

**Supplemental Information _2. Metanalysis**

*PRISMA Diagram*


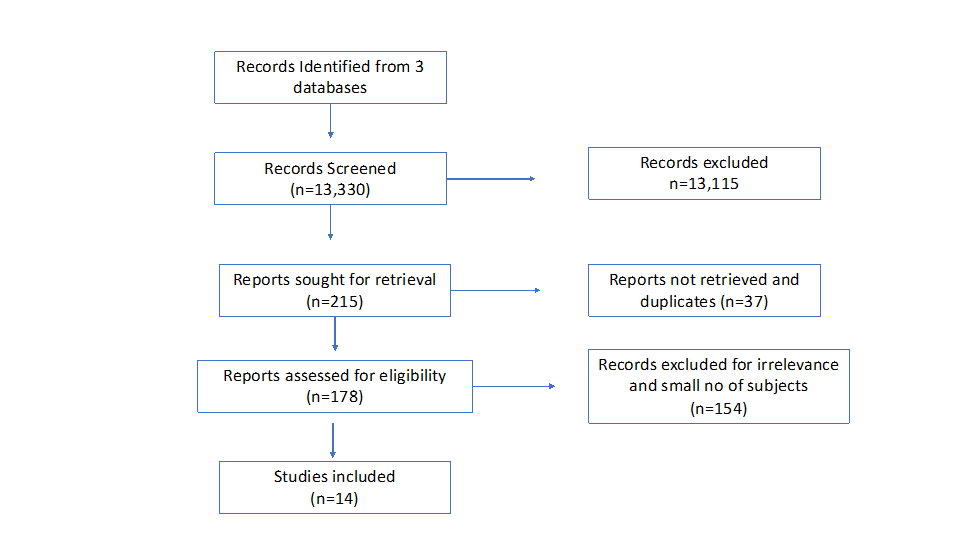


*Metaanalysis for point estimate*


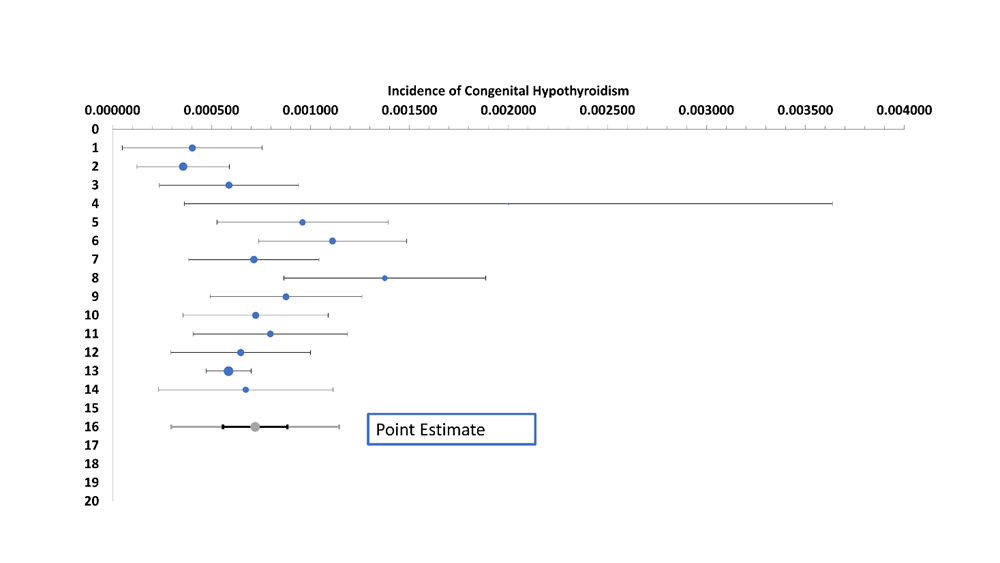


*Egger’s Plot:*


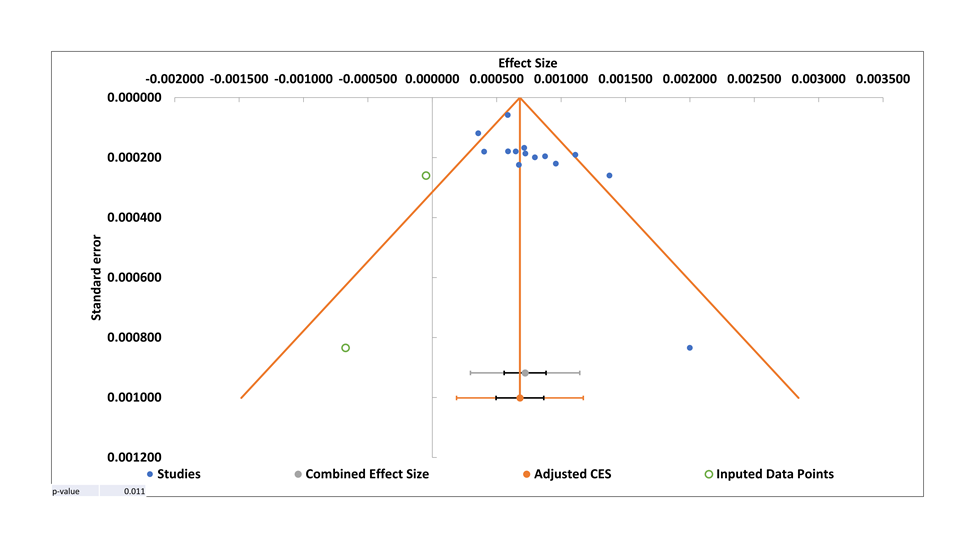

Supplement: Supplementary file 1 [file Data_Sheet_1.docx]
